# Incidence and features of maculopapular rashes in patients receiving immune checkpoint inhibitors^؆^

**DOI:** 10.1016/j.abd.2026.501446

**Published:** 2026-08-06

**Authors:** Gloria Juan-Carpena, Natividad Martínez-Banaclocha, Juan Carlos Palazón-Cabanes, María Niveiro-de Jaime, Isabel Betlloch-Mas, Mar Blanes-Martínez

**Affiliations:** a Department of Dermatology, Morales Meseguer University Hospital, Murcia, Spain; b Toxirel Investigation Group, Alicante, Spain; c Department of Oncology, Dr. Balmis University General Hospital, Alicante, Spain; d Institute for Health and Biomedical Research, Alicante, Spain; e Department of Dermatology, 12 de Octubre University Hospital, Madrid, Spain; f Department of Pathology, Alicante University General Hospital, Alicante, Spain; g Department of Dermatology, Dr. Balmis University General Hospital, Alicante, Spain; h Miguel Hernández University of Elche, Alicante, Spain

Dear Editor,

Maculopapular Rash (MPR) is reported as one of the most frequent cutaneous immune-related Adverse Events (cirAEs) in patients receiving Immune Checkpoint Inhibitors (ICIs).^1^, ^2^, ^3^ However, the incidence, features, and management of MPR in real-world clinical practice remain unclear. We performed a real-world, prospective, observational study in the Department of Dermatology of Alicante University General Hospital (a Spanish tertiary care hospital) including all adults who were starting ICI therapy between 1 March 2020 and 31 May 2022. Clinical trial participants were excluded. Patients attended scheduled follow-up visits (before the first oncological treatment and at months 1, 3, 6 and 12) or urgent consultations when necessary. The minimum follow-up period was 12-months, and surveillance continued until March 2023. We recorded data on the appearance, date of onset, features, type, grade, and management of all cirAEs. However, we specifically describe here the incidence, features, and management of MPR.

We included 189 patients, of whom 82 (43.4%) experienced cirAEs. A total of 107 (56.6%) participants were diagnosed with non-small cell lung cancer, 18 (9.5%) with melanoma, 27 (14.3%) with nephro-urological tumors, and 37 (19.6%) with other neoplasms. Regarding treatment, 71 (37.6%) subjects received pembrolizumab, 43 (22.7%) nivolumab, 1 (0.5%) cemiplimab, 57 (30.2%) atezolizumab, 5 (2.6%) durvalumab, 2 (1.1%) avelumab and 10 (5.3%) the combination of ipilimumab and nivolumab.

Eight participants (4.2%) presented with MPR. Pruritus (35; 18.5%) and eczema (20; 10.6%) were more frequent than MPR, whereas psoriasis (5; 2.6%) and lichen-like reactions (5; 2.6%) occurred less frequently. Other cirAEs, including urticarial reactions (4; 2.1%), folliculitis (3; 1.6%), hidradenitis suppurativa (2; 1.1%), and rosacea, prurigo, fixed drug eruption, mucositis, pseudolymphoma, genital lichen sclerosus, perioral dermatitis, sarcoidosis, and Sweet syndrome-like reactions (1; 0.5% each), were less common.

MPRs were defined as eruptions characterized by confluent erythematous maculopapular lesions with a symmetrical distribution, predominantly involving the trunk and proximal portions of the extremities (Fig. 1, Fig. 2, Fig. 4). Table 1 summarizes participants’ oncologic history and the features and management of MPRs. All participants were males, and the mean age was 66.4 (Standard Deviation [SD] 9.9) years. All were diagnosed with lung cancer (four adenocarcinomas and four squamous cell carcinomas). Half of the participants received pembrolizumab and the other half atezolizumab. The grade of MPR was severe (Grade 3) (Fig. 1, Fig. 4) in four cases and mild (Grade 2) (Fig. 2) in the other four. We identified no relationship between MPR severity and type of ICI. The median latency time was 40.5 (interquartile range 13.5–90.25) days. Five participants (62.5%) had associated pruritus, starting before the MPR in one case. Seven participants underwent a biopsy. The lichenoid pattern (Fig. 3) was the most frequent histologic pattern, appearing in four biopsies (57.1%). A mixed pattern (lichenoid and spongiotic) could be observed in two biopsies (28.6%), and one of them (14.3%) showed a superficial perivascular lymphoid infiltrate. Five participants (62.5%) were prescribed an oral corticosteroid tapering regimen, and one received intravenous corticosteroids (initiated by oncologists). The ICI therapy had to be interrupted in the four patients with severe MPR, but was restarted in all cases after the MPR improved. One participant (12.5%) needed narrow-band ultraviolet-B phototherapy to control the skin toxicity. Six patients (75%) responded to oncologic treatment (stable disease or partial response), and 5 (62.5%) were alive on the study closing date. Three participants (37.5%) experienced at least one other cirAE, and extracutaneous immune-related adverse events were frequent (4 people, 50%).

**Fig. 1:**
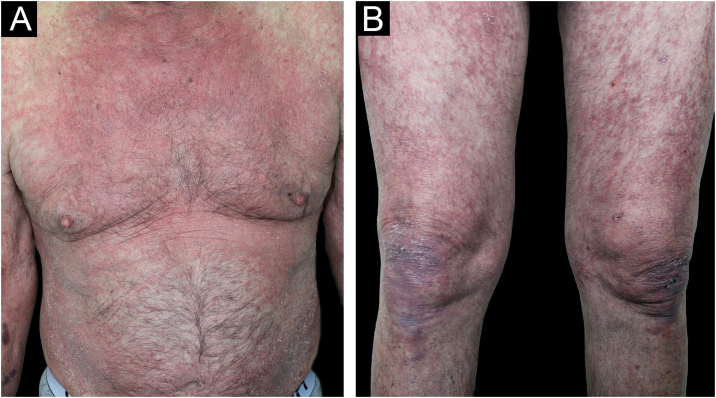
(A‒B) Patient 2. 65-year-old patient with stage IV lung adenocarcinoma presenting with a Grade 3 Maculopapular Rash (MPR): confluent and slightly scaly maculopapular lesions with intense erythema in the trunk and extremities covering > 30% Body Surface Area (BSA).

**Fig. 2:**
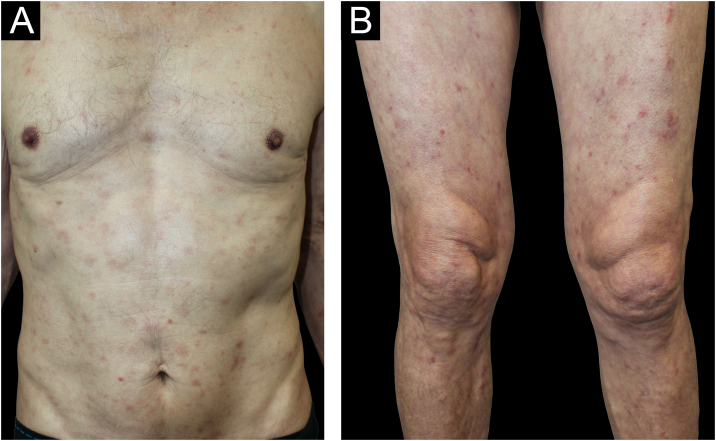
(A‒B) Patient 4. 66-year-old-patient with stage IV lung squamous cell carcinoma presenting with a Grade 2 MPR: Scattered erythematous maculopapular lesions in the trunk and thighs, covering 10%–30% BSA.

**Fig. 4:**
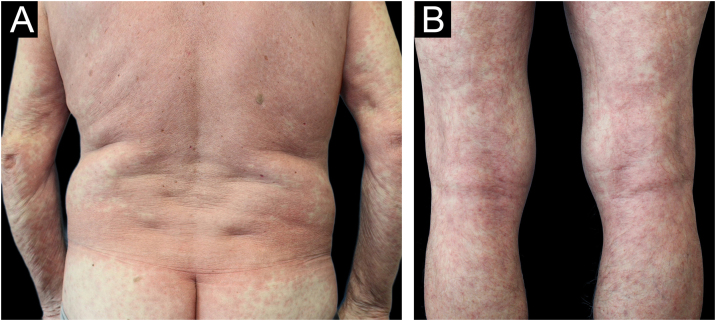
(A‒B) Patient 6. 71-year-old patient with stage IV lung squamous cell carcinoma presenting with a Grade 3 MPR: confluent maculopapular lesions with intense erythema, more pronounced in pressure-dependent areas, covering > 30% BSA.

**Table 1:** Maculopapular Rash (MPR) in patients on Immune Checkpoint Inhibitor (ICI) therapy: oncologic history, MPR features, and MPR management.

Patient	Sex, age	Tumor type and stage	ICI drug	Response	TTO of MPR^a^	Grade	Pruritus	Histology	Treatment	Other irAEs
1	M, 67 yrs	Lung squamous cell carcinoma Stage IV	Atezolizumab (monotherapy)	Initial response to cancer treatment (SD). DP after 8 mths and dead from cancer 15 mths after starting ICI	11 days	3	Yes	_	Antihistamines, high potency TC, oral prednisone Temporary interruption of ICI	Cutaneous: Gr1 acral lichenoid dermatitis, Gr1 oral mucositis
2 (Fig. 1 a‒b)	M, 65 yrs	Lung adenocarcinoma Stage IV	Pembrolizumab (monotherapy)	Almost complete response maintained until study closing date. Alive on study closing date	43 days	3	Yes	Lichenoid interface dermatitis	Antihistamines, high potency TC, oral prednisone Temporary interruption of ICI	Cutaneous: Gr1 xerosis, Gr1 seborrheic dermatitis, Gr1 vitiligo Extracutaneous: Gr1 polyarthralgia
3	M, 45 yrs	Lung adenocarcinoma Stage IV	Pembrolizumab (+carboplatin, pemetrexed)	Nonresponse to cancer treatment. Dead from cancer 6 wks after starting ICI	14 days	2	Yes	Superficial perivascular lymphoid infiltrate	Antihistamines	_
4 (Fig. 2 a‒b)	M, 66 yrs	Lung squamous cell carcinoma Stage IV	Pembrolizumab (+carboplatin, paclitaxel)	PR to cancer treatment. DP after 12 mths of starting ICI. Alive on study closing date	115 days	2	No	Lichenoid interface dermatitis (Fig. 3)	High potency TC, oral prednisone	Extracutaneous: Gr2 autoimmune hepatitis
5	M, 79 yrs	Lung adenocarcinoma Stage IV	Atezolizumab (monotherapy)	Nonresponse to cancer treatment. Dead from cancer 5 wks after starting ICI	38 days	2	No	Lichenoid interface dermatitis	Antihistamines, high potency TC, IV prednisone	_
6 (Fig. 4 a•b)	M, 71 yrs	Lung squamous cell carcinoma Stage IV	Atezolizumab (monotherapy)	Initial response to cancer treatment (SD). DP after 9 mths of starting ICI. Alive on study closing date	12 days	3	Yes	Spongiotic and lichenoid interface dermatitis (mixed pattern)	Antihistamines, high potency TC, oral prednisone Temporary interruption of ICI	Extracutaneous: Gr2 pneumonitis
7	M, 73 yrs	Lung squamous cell carcinoma Stage III	Pembrolizumab (monotherapy)	Initial PR to cancer treatment. DP after 10 mths of starting ICI. Alive on study closing date	115 days	3	Yes	Spongiotic and lichenoid interface dermatitis (mixed pattern)	Antihistamines, high potency TC, oral prednisone, NB-UVB phototherapy Temporary interruption of ICI	Cutaneous: Gr3 pruritus (starting before the MPR)
8	M, 65 yrs	Lung adenocarcinoma Stage IV	Atezolizumab (monotherapy)	Initial response to cancer treatment (SD). DP after 4 mths of starting ICI. Alive on study closing date	82 days	2	No	Lichenoid interface dermatitis	High potency TC	Extracutaneous: Gr1 diarrhea. Gr1 fever

DP, Disease Progression; Gr, Grade; ICI, Immune Checkpoint Inhibitor; irAEs, Immune related Adverse Events; IV, Intravenous; M, Male; MPR, Maculopapular Rash; mths, months; NB-UVB, Narrowband Ultraviolet B; PR, Partial Response; SD, Stable Disease; TC, Topical Corticosteroid; TTO, Time To Onset; yrs, years; wks, weeks.

a Mean time to MPR onset or mean latency time was 53.8 (standard deviation 44.4) days, and median time to MPR onset or median latency time was 40.5 (interquartile range 13.5–90.25) days.

**Fig. 3:**
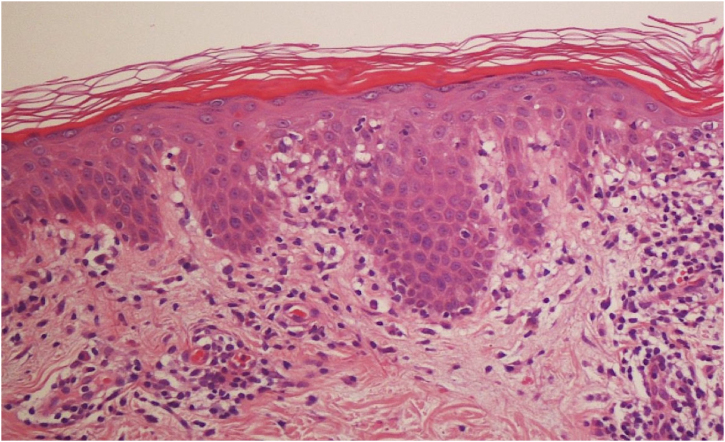
Histology image of biopsy taken from patient 4 (Hematoxylin & eosin, 10×). Lichenoid interface dermatitis; moderate lymphoid infiltrate in the upper dermis with interface damage in the basal epidermis and necrotic keratinocytes.

There are no previous series in the literature that specifically describe MPR in patients treated with ICI, so the epidemiologic characteristics and oncologic history of this population are unclear. In our real-world prospective study, MPRs occurred only in men with lung cancer. The cumulative incidence of MPRs was lower in our study than in most other reports. Previous estimates (10%‒45%) have probably overstated the real values owing to possible selection biases and use of confusing terms in retrospective studies,^1^, ^2^, ^3^, ^4^, ^5^ or the use of the generic term “rash” to name different types of cutaneous toxicities in clinical trials.^6^ The clinical presentation in our population was similar to that formerly described in the literature. However, Grade 3 rashes were more common, since severe MPRs have previously been reported in less than 3%.^1^, ^2^ The most frequent histologic pattern observed in our patients was not the spongiotic pattern, as formerly reported,^7^ but the lichenoid interface pattern. Although a lichenoid interface pattern may be found in different types of cirAEs, including MPRs, we consider that the term “lichen-like reactions” should be reserved for eruptions fulfilling both characteristic clinical features (violaceous papules in typical locations such as the wrists and ankles, Wickham striae, post-inflammatory hyperpigmentation, mucosal involvement) and histological lichenoid changes.

The management of our patients was in line with recommendations from Apalla et al.;^2^ accordingly, there was no need to discontinue ICI permanently in any case. When MPR develops, it is important to rule out other immune-related adverse events. People with MPR are likely to respond well to ICI therapy, as previously described for other cirAEs.^8^, ^9^, ^10^

## ORCID ID

Natividad Martínez Banaclocha: 0000-0002-7515-5037

Juan Carlos Palazón-Cabanes: 0000-0002-3936-2362

María Niveiro-de Jaime: 0000-0003-1507-7847

Isabel Betlloch-Mas: 0000-0003-0050-6178

Mar Blanes-Martínez: 0000-0003-2886-7493

## Ethical approval

This study was approved by the local Institutional Review Board (reference numbers PI2021-003; 2020-0323).

## Ethics statement

The patients in this manuscript have given written informed consent for the publication of their case details.

## Data availability statement

The data that support the findings of this study are available from the corresponding author upon reasonable request.

## Financial support

This project received a research grant of EUR 1000 from the Alicante Institute for Health and Biomedical Research, Spain (ISABIAL), to help cover the costs of statistical analysis and translation (reference number 2020-0323).

## Authors’ contributions

Gloria Juan-Carpena: Approval of the final version of the manuscript; critical literature review; data collection, analysis and interpretation; intellectual participation in propaedeutic and/or therapeutic management of studied cases; preparation and writing of the manuscript; statistical analysis; study conception and planning.

Natividad Martínez Banaclocha: Approval of the final version of the manuscript; data collection, analysis and interpretation; effective participation in research orientation; manuscript critical review; study conception and planning.

Juan Carlos Palazón-Cabanes: Approval of the final version of the manuscript; data collection, analysis and interpretation; intellectual participation in propaedeutic and/or therapeutic management of studied cases.

María Niveiro-de Jaime: Approval of the final version of the manuscript; intellectual participation in propaedeutic and/or therapeutic management of studied cases.

Isabel Betlloch-Mas: Approval of the final version of the manuscript; manuscript critical review; study conception and planning.

Mar Blanes-Martínez: Approval of the final version of the manuscript; data collection, analysis and interpretation; effective participation in research orientation; intellectual participation in propaedeutic and/or therapeutic management of studied cases; manuscript critical review; statistical analysis; study conception and planning.

## Research data availability

The entire dataset supporting the results of this study was published in this article.

## Conflicts of interest

None declared.
